# Towards an Optimization of Stimulus Parameters for Brain-Computer Interfaces Based on Steady State Visual Evoked Potentials

**DOI:** 10.1371/journal.pone.0112099

**Published:** 2014-11-14

**Authors:** Anna Duszyk, Maria Bierzyńska, Zofia Radzikowska, Piotr Milanowski, Rafał Kuś, Piotr Suffczyński, Magdalena Michalska, Maciej Łabęcki, Piotr Zwoliński, Piotr Durka

**Affiliations:** 1 University of Social Sciences and Humanities, Warsaw, Poland; 2 University of Warsaw, Faculty of Physics, Warsaw, Poland; 3 Nencki Institute of Experimental Biology PAS, Warsaw, Poland; 4 Warsaw Memorial Child Hospital, Department of Neurosurgery, Warsaw, Poland; Georgia State University, United States of America

## Abstract

Efforts to construct an effective brain-computer interface (BCI) system based on Steady State Visual Evoked Potentials (SSVEP) commonly focus on sophisticated mathematical methods for data analysis. The role of different stimulus features in evoking strong SSVEP is less often considered and the knowledge on the optimal stimulus properties is still fragmentary. The goal of this study was to provide insight into the influence of stimulus characteristics on the magnitude of SSVEP response. Five stimuli parameters were tested: size, distance, colour, shape, and presence of a fixation point in the middle of each flickering field. The stimuli were presented on four squares on LCD screen, with each square highlighted by LEDs flickering with different frequencies. Brighter colours and larger dimensions of flickering fields resulted in a significantly stronger SSVEP response. The distance between stimulation fields and the presence or absence of the fixation point had no significant effect on the response. Contrary to a popular belief, these results suggest that absence of the fixation point does not reduce the magnitude of SSVEP response. However, some parameters of the stimuli such as colour and the size of the flickering field play an important role in evoking SSVEP response, which indicates that stimuli rendering is an important factor in building effective SSVEP based BCI systems.

## Introduction

Individuals with neuromuscular disorders such as multiple sclerosis, amyotrophic lateral sclerosis, and locked-in syndrome have no voluntary control of their muscles and are often unable to communicate. Brain Computer Interface (BCI) systems give them an opportunity to have contact with the external world and accomplish simple, everyday activities. BCI systems are most frequently based on recordings of the brain's electrical activity from the scalp (electroencephalogram, EEG) because of the relatively low price and portability. In this study we investigated a BCI system based on the Steady State Visual Evoked Potentials (SSVEP) phenomenon.

SSVEPs can be detected mainly in EEG signals recorded from above the visual areas of the scalp as a response to stimulation with light flickering with fixed frequency [1]. During such stimulation, increases in EEG power at the frequency of stimulation can be observed. SSVEPs are detected at stimulus frequency, its harmonics and subharmonics [2]. The SSVEP spectrum shows characteristic peaks which are relatively stable over time [3], [2]. Stimuli eliciting SSVEP can be characterized by different properties which affect the strength of the response, like colour and shape.

Perception of visual stimuli depends on characteristics of the human nervous system. In order to explain presented results we need first to describe briefly the operating principles of the visual system, because its features influence the processing of particular stimuli. SSVEP generation is as an outcome of stimulation repeated with certain frequency, so motion perception seems to be the prime to generate this type of response.

The human visual system consists of three parallel information processing pathways: Parvocellular (PC), Magnocellular (MC), and Koniocellular (KC) [4]. Each of them is responsible for processing specific physical parameters of the stimulus and is characterized by different temporal and spatial resolutions (see: [4], [5]). The magnocellular pathway originates from L and M cones in the retina. It is sensitive to differences in achromatic contrast and motion [6], and carries information about depth [4]. The receptive fields of the MC pathway are relatively large [7] and exhibit a transient response to changes in retinal stimulation, which begins and ends quickly [8]. The PC pathway mainly carries information about colour (red and green) and shape [9]. Receptive fields of this pathway are typically half the size of magnocellular fields [4] and exhibit a more sustained response to changes in retinal stimulation [8]. The KC pathway carries information about blue and yellow colour and reacts to spectral stimuli [10]. Visual pathways play a crucial role in the formation of SSVEPs at the cortical level. We expected that stimuli processed by the MC pathway (e.g. brighter and larger), which is responsible for perception of motion, would evoke the biggest SSVEP amplitude.

Colour seems to be the most evident feature to be examined, because the visual pathways process different colours in different ways. Experiments performed by Regan in 1966 (see also [1]) showed that blue, red, and yellow stimuli presented at certain frequencies evoke SSVEPs with different magnitudes. Red stimuli gave the strongest response in 11 Hz, while blue stimuli were less sensitive to frequencies and gave the strongest response in 13 Hz. SSVEP elicited by yellow stimuli was least dependent on frequency and gave the lowest response. An impact of frequency and different colour interaction was shown by Gerloff [11]. A checker-board with different combinations of hues and flickering with frequencies ranging from 6 to 17 Hz was used to evoke SSVEPs. However, results of this study do not allow for inference on the relation of stimulation colour to amplitude of SSVEP and are characterized by large intra- and inter-subject variability.

A review of 59 papers written by Zhu in 2010 [12] indicates that green, black, gray, red, and white are currently the most commonly used stimuli colours in SSVEP-based BCIs. However, it is not known which of these colours is best for SSVEP-based BCIs, as none of these experiments directly investigated the influence of colour on strength of the SSVEP response.

Knowledge about the influence of stimulus size on SSVEP response seems to be crucial in the design of graphic user interfaces, because the size of a single flickering field determines the number of simultaneously presented stimuli. Another important parameter of stimuli used in SSVEP-BCI's is the distance between flickering fields. Knowledge about the influence of these parameters on brain response is crucial for an optimal design of BCI systems.

As for the shapes and patterns of the stimuli, Zhu [12] concluded that checkerboards, squares, and rectangles are the most common in BCI-related studies. However, author concludes that no general conclusion can be drawn about their influence on the strength of SSVEP response. In an experiment from 2007 [13], plain stimuli gave stronger SSVEP response than checkerboards and striped stimuli. Due to different shapes of receptive fields in successive stages of information processing in the visual system, one can hypothesize that square stimuli will evoke better response than circular ones [14]. Spatial attention is another factor that can influence the SSVEP response. Amplitude of the response can change as a function of the user's concentration on the stimulus [15], [16], [17]. It is generally assumed that presence of a fixation point minimizes undesired eye movements and helps users to concentrate on the chosen stimulus [18]. Environmental conditions should also be considered; for example it was shown that a darkened room has positive influence on the strength of the SSVEP response [19].

On the other hand, it seems that a stimulus evoking strong SSVEP response in particular single trial is not identical to the most optimal stimulus in BCI systems. A selection of stimulus parameters to BCI systems ought to take into account both the physiological and psychological processes. It is known that high intensity stimulus evokes the strongest response of sensory systems. However, the stronger stimulus is perceive, the faster a user gets tired and the weaker focus of attention becomes. It seems that a compromise to both point of view: to maximize a strength of cerebral response and minimalize a fatigue and displeasure. Based on physiological research we hypothesized that the big and fair stimuli evoke magnitude of SSVEP, but we were interested in whether in case of long and tiring stimulation less aggressive stimuli give better results.

Overall, the existing state of the art does not clarify which choices of stimuli features are the best for SSVEP-based BCIs. Nevertheless, many studies conclude that experimental design and paradigm are crucial in developing efficient BCI systems [5], [20].

In this study we investigate the parameters of stimuli, which positively affect the magnitude of SSVEP response measured by EEG. The experimental paradigm was designed to simulate a real BCI system as close as possible. Block of trials lasted ∼45 minutes, which is a period of time sufficient to write a short massage by potential BCI-user. The goal was to measure the strength of the SSVEP response related to parameters of the stimuli as well as to the psychological factors such as focus of attention, motivation and tiredness. In two experiments we systematically measure the SSVEP response to stimuli with varying parameters, including colour, size, shape, inter-stimulus distance, and presence or absence of a fixation point.

## Materials and Methods

Results presented in this paper come from two consecutive experiments. Experiment I was a test of five stimulus parameters that could potentially influence the magnitude of SSVEP response over a relatively wide range of their values. Based upon its results, three parameters with narrowed ranges were chosen for the second experiment conducted on a larger group of subjects.

### 1. Participants

In Experiment I, five young adults (*M_age_* = 25.8; SD  = 1.79) of both sexes were examined. 20 subjects participated in Experiment II (*M_age_* = 27.2; SD  = 3.3). All subjects were screened for photogenic epilepsy, neurological and psychiatric disorders, and use of medications known to adversely affect EEG recording. No financial compensation was given. All participants were informed about the experiment procedure and signed a written consent.

### 2. Experimental setup

Both experiments were carried out in a darkened room with windows curtained. Two desk lamps were the only light sources. Subjects were sitting on a chair one meter from the center of the display. Experiments were divided into sessions. Lengths of the breaks between sessions were controlled by the participant. Each session lasted 45 minutes and each trial included 4 seconds of stimulation and a 6 second rest period. Each of the presented stimuli was repeated 30 times.

**Figure 1:**
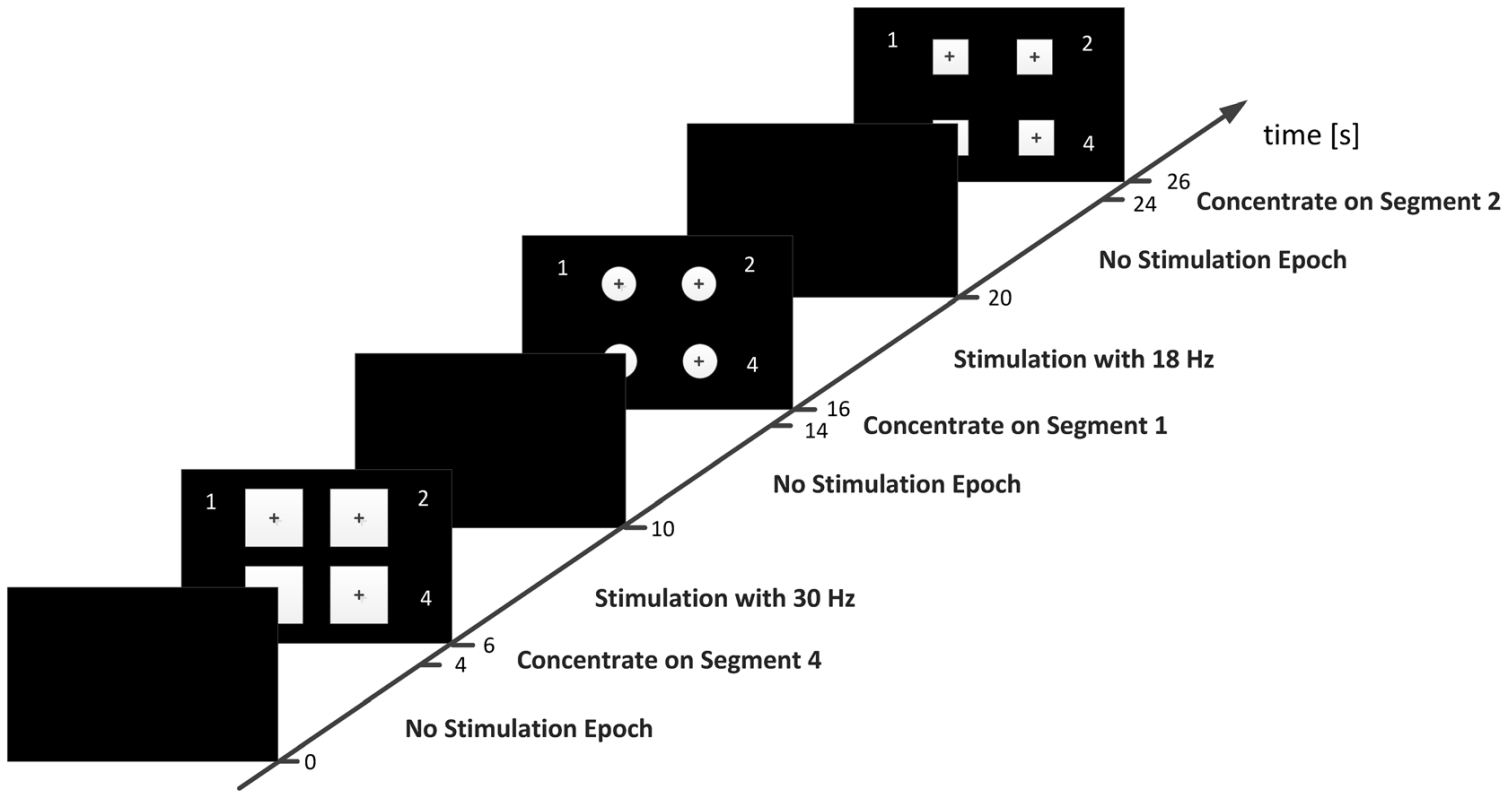
Time course of the experimental paradigm.

Four stimuli were presented simultaneously and subjects were asked to concentrate on the one indicated by an auditory cue. The schematic sequence of events is presented in Fig. 1. Experiment I consisted of 4 s long stimulation periods interleaved by 6 s long resting periods. The screen was black during the rest period. In order to create experimental conditions corresponding to the SSVEP paradigm used in BCI systems, all four fields were simultaneously active (each flickering at a different frequency) during the stimulation intervals. Four frequencies of stimulation (14, 17, 25, and 30 Hz) were chosen on the basis of the results obtained by Kuś [21]. Investigated parameters (colour, size, etc.) were software controlled and randomly presented on an LCD screen, while the flickering was generated by the underlaid LEDs. Stimuli were presented on a hybrid device [22] constructed at the Faculty of Physics, University of Warsaw in order to optimize stability of stimuli rendering. The device consists of an array of LEDs underlaid below an LCD screen (195 mm high and 350 mm wide), where the LEDs highlight precisely determined area of the screen. Each of the four squares displayed on the LCD screen is highlighted by a group of LEDs, flickering with frequencies controlled by the software. Using such a device eliminates problems with monitor refresh rate and at the same time enables full control of stimulus appearance.

### 3. Experiment I

The first experiment was designed to investigate the influence of five parameters — shape, colour, distance between stimuli, size, and presence or absence of the fixation point — on the magnitude of SSVEP response. Four sizes (angular size in degrees) were investigated: ∼0.57°, ∼1.49°, ∼2.6°, and ∼3.72. Stimuli were organized in three different inter-stimulus distance settings: next to each other (no distance  = 0°), centered on each stimulus area (medium distance  =  ∼2.3°), and on the opposite points of the presentation area (long distance  =  ∼4.93°). The five examined colours were chosen from the RGB model: blue, red, green, white, and yellow. The luminance of white and yellow stimuli was 30 lx, green - 20 lx, red - 12 lx, blue – 4 lx, and black background - 2 lx. Two stimuli shapes were used: square and circle (both had equal surface areas). The absence and presence of a fixation point located in the middle of each flickering field was also examined. A detailed description of the investigated stimuli is listed in Table 1. This experiment consisted of six sessions of 45 minutes each.

**Table 1:** Parameters of the stimulus used in Experiment I.

Tested parameter	Dimension	Inter-stimulus distance	Fixation point	Stimulus colour	Shape
Size	∼0.57°, ∼1.49°, ∼2.60°, ∼3.72°	∼4.76°, ∼3.50°, ∼2.30°, ∼1.26°	yes	white	square
Inter-stimulus distance	∼2.60°	0° , ∼2.30°, ∼5.27°	yes	white	square
Colour	∼2.60°	∼2.30°	yes	white, red, green, yellow, blue	square
Absence of fixation point	∼2.60°	∼2.30°	no	white	square
Shape	∼2.60°	∼2.30°	yes	white	circle, square

### 4. Experiment II

Based upon the results of Experiment I, we chose three parameters for further investigation in Experiment II and conducted it on a larger population using more restricted ranges of variability. We did not further investigate the shape and inter-stimulus distance, because these parameters showed no significant influence on the response in Experiment I. We restricted the variability of remaining parameters to the following ranges: colours yellow, white, and red, sizes ∼2.6° and ∼3.72°. Additionally presence/absence of fixation point was tested due to participants' suggestion that it had helped them to concentrate This parameter was a substitute of signs which are located in the flickering field in real BCI systems. Detailed parameters of selected stimuli are given in Table 2. Presented stimuli were circular as this shape evoked slightly stronger SSVEP-response; however, this difference was not statistically significant.

**Table 2:** Parameters of the stimulus used in Experiment II.

Tested parameter	Dimension	Inter-stimulus distance	Fixation point	Stimulus colour	Shape
Size	∼2.60°, ∼3.72°	∼2.30°, ∼1.26°	yes	white	circle
Colour	∼2.60°	∼2.30°	yes	white, red, yellow	circle
Absence of fixation point	∼2.60°	∼2.30°	no	white	circle

### 5. Data acquisition

The EEG data acquisition was performed using the EasyCap EEG positioning system and a 32-channel Porti 7 amplifier made by TMSI. It was connected to the computer via a USB interface using optical fiber. The scalp area was prepared before placing the electrodes and conductive gel was used in order to reduce skin impedance.

The data was recorded with a 1024 Hz sampling rate. Skin impedance was maintained below 5k Ohms. 20 electrodes were used. 19 electrodes were placed in a 10–20 system and there was one additional electrode FCz. Averaged signal from mastoids (M1 and M2 electrodes) was used as a reference. The ground electrode was placed on the chest near the breastbone area. Dedicated software was used for data acquisition and stimuli presentation. This software is available on terms of the GPL license from http://git.braintech.pl and http://braintech.pl/svarog.

### 6. Ethics statement

The project was approved by the Research Ethics Committee at University of Social Sciences and Humanities in Warsaw, Poland. All participants declared the absence of neurological and mental illnesses, and were screened against the photosensitive epilepsy with the standard clinical EEG test. Informed, written consent was obtained from all of the participants.

### 7. Data analysis

#### 7.1. Signal pre-processing

Seven channels from occipital and parietal areas were chosen for analysis: O1, O2, Pz, P3, P4, P7, and P8, all down-sampled to 128Hz. Placement of these electrodes corresponds to primary (O1 and O2) and secondary visual areas, thus the signal collected from these areas should be the most significant in terms of SSVEP response energy (Pastor, 2003). Downsampling was conducted using a Chebyshev type I filter of order 8. Next, from specified channels, two classes of segments were extracted: 4s long epochs of signal recorded during the visual stimulation with frequency *f* denoted as 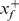 and 4s long epochs measured before the onset of stimulation with frequency *f*, marked as 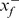.

#### 7.2. Frequency domain filtering

We expected that the most prominent changes in EEG signal during the visual stimulation would be observed at the stimulation frequency. Therefore, all segments in both classes were band-pass filtered by means of a 3rd order elliptic filter with pass-band centered at the given frequency stimulation *f*. The width of the pass- band was 2 Hz. The level of the filter peak-to-peak ripple in the pass-band was 0.04 dB, whereas the minimum stop-band attenuation was 40 dB. The filtered time series are denoted as 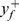 and 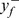.

#### 7.3. Spatial filtering

It is important to combine information carried in analyzed channels to estimate the SSVEP response. Analyzing each channel separately can be misleading, as the SSVEP changes significantly not only from subject to subject but also as far as topology is concerned. This means that to observe the SSVEP, one should take into account several electrodes at once. To estimate the montage of EEG, which amplifies the magnitude of the SSVEP response, we used the Common Spatial Patterns (CSP) method. CSP estimates a spatial filter, that is, a linear combination of channels, which is optimal for discrimination between two different experimental conditions (for a full method description see [23], [24], [25]) in terms of variance. Here the signals 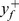 and 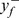
were used to set the CSP filter for each stimulation frequency *f* separately. Applying the CSP filter to original signals from both classes 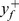 and 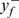 results in two one-dimensional signals 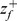 and 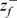 which differ mostly in terms of variance. The signal 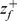 has a large variance when there was a response (at a given frequency) and 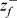 has a small variance when no response was present.

#### 7.4. Measures of SSVEP response

The estimation of magnitude of SSVEP was performed in two steps:

Assessment of the power spectrum 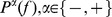
of the signals 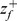 and 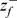 was conducted using the Welch method with Hanning window of 1 s length with a 3/4 second overlap. It is known that the spectral power of EEG decreases as the frequency increases. This property implies that response to high-frequency SSVEP has lower absolute power than response to low-frequency stimulation. Therefore, SSVEP strength can be better measured as a relative increase of power at the stimulation frequency or its harmonic, in respect to its baseline value (spontaneous EEG activity). The quantity, which measures the relative increase or decrease of EEG power in Event Related Spectral Perturbation [26], is defined as follows:
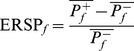
where 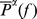
is the 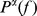 averaged over experiment realizations with given stimulation frequency *f*. ERSPs reflect stimulus-induced changes in spectral power within a particular frequency band.

#### 7.5. Statistical inference

The statistical significance of difference for estimated ERSPs in each tested group was calculated using the Friedman test [27]. For every subject, each tested group consisted of ERSPs determined for one frequency. If the test indicated a statistically significant difference, a post-hoc Wilcoxon signed-rank [28] test was performed to check which condition differed from others. During these calculations, a single hypothesis was tested multiple times, therefore we applied the Bonferroni correction to account for multiple comparisons, dividing the significance level by the number of comparisons done in each group [29].

## Results

This section presents the results of statistical analysis of differences in the magnitude of SSVEP response elicited by different stimuli. As described in the „Data Analysis” section, the magnitude of the response was quantified as the relative change of spectral power in the corresponding frequency band and calculated after applying the optimal common spatial filter (see Section „Data Analysis”). Both of these analysis methods are commonly used in SSVEP-based BCI systems, so the presented results can be directly compared and applied to BCI systems.

There were 30 repetitions of recorded SSVEP response for each subject and each combination of stimuli parameters and frequency. This allowed for a separate assessment of the statistical significance of changes for each subject. Investigation of inter-subject variability was beyond the scope of this study, so we decided to concentrate on the mean effects. All of the results presented in this section were obtained by pooling together measurements for each combination of stimuli parameters and frequencies obtained from all of the subjects.

### 1. Effect of colour on magnitude of SSVEP response

**Figure 2:**
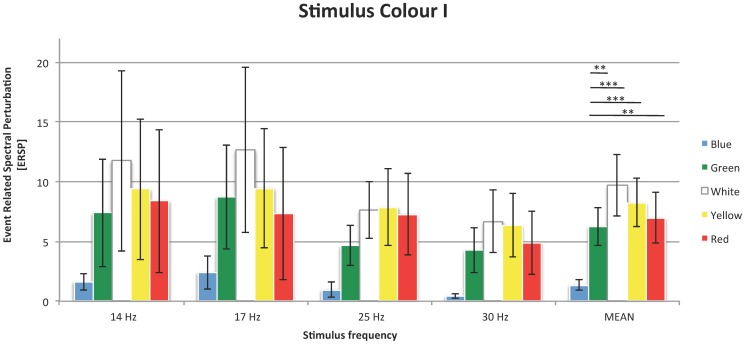
Relative power increase for SSVEP response to different colours of stimuli. Mean computed for all the subjects. Error bars indicate standard error of the mean (SEM). Horizontal lines above the bars indicate statistically significant differences on the level of p<0.001 (***), p<0.01 (**), and p<0.05 (*).

The impact of colours presented in Experiment I on mean SSVEP was examined using the Friedman test, indicating significant differences across all frequencies (*χ* ^2^(4)  = 29.96; *p*<0.001). Mean ERSP for all participants (Fig. 2) showed that the weakest response is evoked by blue stimuli.

Decrease of SSVEP for stimulation with blue squares was significant in comparison with all other colours. Differences between other colours were not significant in tests conducted on mean results for all subjects. The results of statistical analysis are shown in Table 3.

**Table 3:** The results of post-hoc Wilcoxon signed-rank for comparison of SSVEP magnitude pairs evoked by different coloured stimuli in experiment I.

	yellow	blue	red	green	white
yellow	-	χ2(1) = 19,24; p<0,001	in.	in.	in.
blue	χ2(1) = 19,24; p<0,001	-	χ2(1) = 11,88; p<0,01	χ2(1) = 13,36; p<0,01	χ2(1) = 19,25; p<0,001
red	in.	χ2(1) = 11,88; p<0,01	-	in.	in.
green	in.	χ2(1) = 13,36; p<0,01	in.	-	in.
white	in.	χ2(1) = 19,25; p<0,001	in.	in.	-

**Figure 3:**
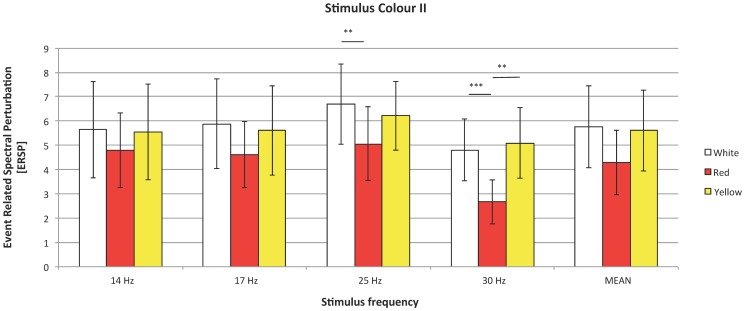
SSVEP responses to stimuli of different colours; organization of the plot as in **Figure 2**.

In Experiment II, differences between yellow, white, and red stimuli were tested (Fig. 3). The Friedman test did not reveal statistically significant results: *χ* ^2^(2)  = 4.44; in.

### 2. Effect of stimulus size on magnitude of SSVEP response

**Figure 4:**
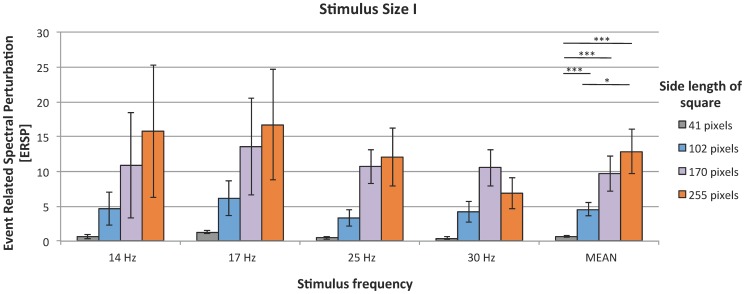
SSVEP responses to stimuli of different sizes; organization of the plot as in **Figure 2**.

In Experiment I, stimulus size examination showed a strong linear effect on SSVEPs (Fig. 4). This was confirmed using the Friedman test performed on mean values for each subject (*χ* ^2^(3)  = 43.81; *p*<0.001). The relative power increased with the size of the stimulus. Post hoc comparisons revealed significant differences between SSVEP magnitudes when side length of 41 pixels was compared to the three other sizes (detailed results of post hoc tests are given in Table 4).

**Table 4:** The results of post-hoc Wilcoxon signed-rank for comparison of SSVEP magnitude evoked by different sized stimuli in experiment II.

	∼0.57°	∼1.49°	∼2.60°4	∼3.72°
∼0.57°	-	χ2(1) = 18,34; p<0,001	χ2(1) = 26,19; p<0,001 in.	in. χ2(1) = 26,19; p<0,001;
∼1.49°	χ2(1) = 18,34; p<0,001	-	in.	χ2(1) = 8,55; p<0,05
∼2.60°	χ2(1) = 26,19; p<0,001 in.	in.	-	in.
∼3.72°	χ2(1) = 26,19; p<0,001; in.	χ2(1) = 8,55; p<0,05	in.	-

**Figure 5:**
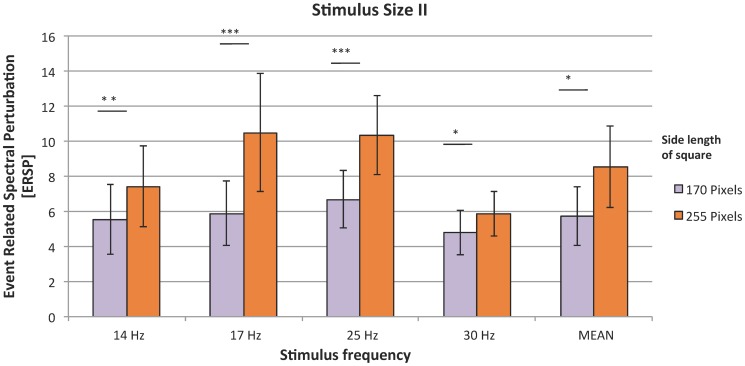
SSVEP responses to stimuli of different sizes; organization of the plot as in **Figure 2**.

In Experiment II, Friedman tests performed for all frequencies confirmed a significant effect of size (*χ* ^2^(1)  = 5.16; p<0.05). Larger stimuli induced higher SSVEP response (mean ERSP  = 8.53) for all frequencies as compared to the response evoked by smaller stimuli (mean ERSP  = 5.76) (Fig. 5).

### 3. Effect of fixation point absence on magnitude of SSVEP response

**Figure 6:**
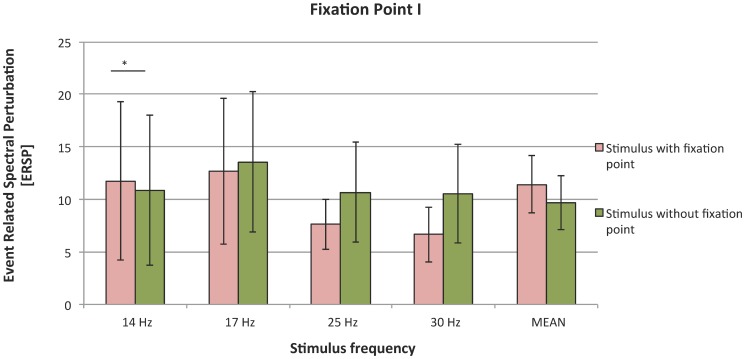
SSVEP responses to stimuli with and without fixation point; organization of the plot as in **Figure 2**.

The mean value of SSVEPs decreased to 9.7 when a fixation point was used during stimuli presentation, compared to 11.4 for stimuli presented without a fixation point (Fig. 6). However, Friedman tests performed on all frequencies indicated that this difference was not significant (*χ* ^2^(1)  = 0.27; in.). Nevertheless, presence of fixation point was taken into consideration in further analysis, because participants reported that it had helped them to concentrate on given field; also, presence of the fixation point corresponds to signs (i.e. letters) appearing in real BCI systems.

**Figure 7:**
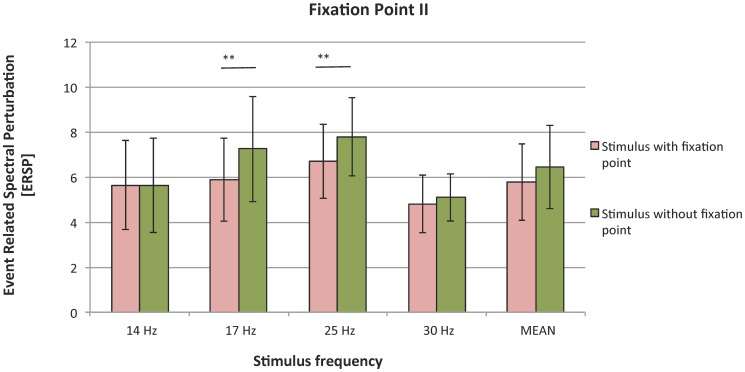
SSVEP responses to stimuli with and without fixation point; organization of the plot as in **Figure 2**.

In Experiment II, larger SSVEP response was visible for stimuli without a fixation point (mean ERSP  = 6.43) than with one (5.76) (Fig. 7). However, comparisons performed across all frequencies revealed no effect of this parameter.

### 4. Effect of stimuli shape on the magnitude of SSVEP response

**Figure 8:**
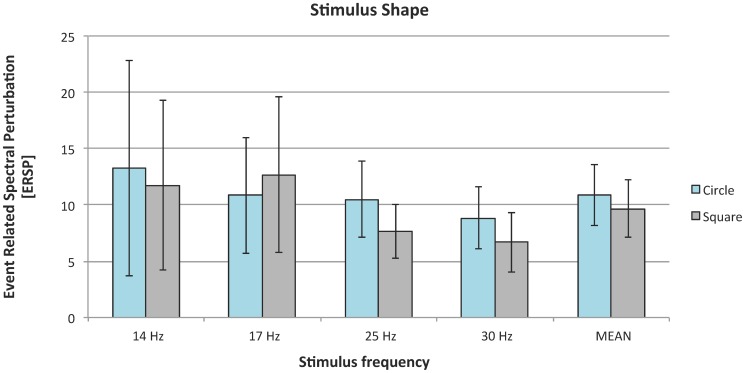
SSVEP responses to circle and square stimuli; organization of the plot as in **Figure 2**.

Shape did not significantly affect SSVEP magnitude: *χ* ^2^(1)  = 0.39; in. Although the effect of shape was not significant, circles evoked higher SSVEP amplitude than squares (Fig. 8). Accordingly, we used this shape in the second experiment.

### 5. Effect of distance between stimuli on magnitude of SSVEP response

**Figure 9:**
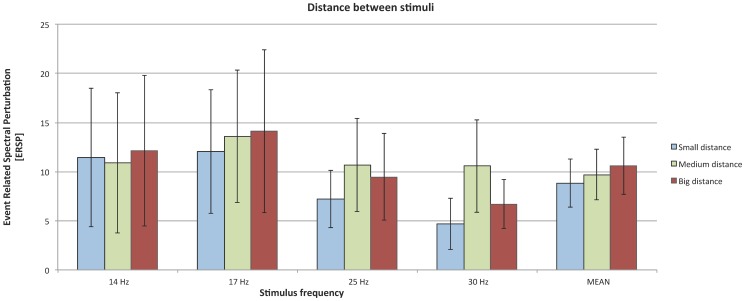
SSVEP responses to circle and square stimuli; organization of the plot as in **Figure 2**.

The influence of distance between stimuli on SSVEP magnitude was examined among all frequencies using Friedman tests, indicating no significant differences: *χ* ^2^(2)  = 1.52; in. (Fig. 9)

## Discussion

To the best of our knowledge, this experiment was the first complex study on the optimization of stimuli features used in SSVEP based BCI systems. In contrast to numerous studies regarding data analysis and technical aspects of BCI system operation, there are few articles on the relevance of stimulation parameters. On the basis of neurophysiological knowledge it can be expected that proper choice of the features of the flickering field could strengthen neural response and ease response detection. This in turn could improve efficiency of SSVEP-based BCIs. On the other hand, the balance between the strong neural response and usability (not tiring, comfortable conditions of long stimulation) seems to be necessary. The existing state of art does not unequivocally answer which choices of stimuli features are best for SSVEP based BCIs. Nevertheless, some authors claim that experimental design and paradigm are crucial for developing efficient BCI systems [5], [20]. In the current study we decided to investigate parameters of the stimuli affecting SSVEP response. Five parameters were investigated for SSVEP based BCIs: colour, size, shape, fixation point presence, and inter-stimulus distance, of which three showed an influence on the strength of SSVEP response. Finding the best stimulus parameters for BCI systems was the main goal of this study. We put a great effort into designing an experimental procedure as similar as possible to BCI. In particular, to include possible interferences between frequencies appearing simultaneously in real BCI systems, we applied four simultaneously flickering fields with different frequencies.

Due to their basic functions (e.g. temporal and spatial resolution), it is suspected that the three visual pathways play a crucial role in SSVEP formation. However, their role is still not well identified [2]. Researchers presume that the strength of SSVEP depends on the cortical location and stimuli appearance [30], [5]. Presented results indicate that every set of parameters evokes SSVEP response, but the largest amplitude was elicited after stimulation characterized by qualities that are processed by the MC pathway (e.g., large plain stimulus with bright colour). One of the tested parameters, which significantly improved the SSVEP response, was the colour of flickering square. Five different colours were used: white, green, red, blue, and yellow, with a black background. We put great effort into the applicability of chosen stimuli in BCI technology. For that reason the selection of stimuli hues and their luminance was based on the RGB model. Stimulation with white and yellow light evoked the largest amplitude. Different colours caused differences in luminance and contrast of the stimulation - white and yellow were the brightest. The obtained results could be explained by the contrast response function [31], which shows the impact of contrast value on V1 neuron firing rates. According to Albrecht [31] the magnitude of neuron activity exponentially increases as the contrast intensifies. In the case of a high contrast, the response saturates. Assuming that the results were an effect of brightness on the strength of SSVEP response, increase of amplitude might be explained by the properties of the MC pathway. One of its characteristics is high sensitivity to luminance changes of perceived objects or better perception of brighter stimulus as compared to the PC pathway [32]. Considering colour as a variable differentiating stimulus, it may be difficult to point to one hypothesis. The MC pathway is not sensitive to changes of hues [32], [9]. The PC pathway is responsible for detection and processing of information about perceived light wavelength, but its temporal resolution is approximately five cycles per second [33], [4]. It can be suspected that dark colours (blue, green) evoke lower SSVEP response in comparison to bright colours (white, yellow) because of the contrast between stimuli and the background (black) [6], [34]. The contrast was not fixed, so bright stimuli were easier to differentiate from the background.

The next parameter tested in this study was the size of the stimulus. The largest square was most effective for SSVEP - based BCI, as it evoked the highest response amplitude. These results are in line with a visual evoked potentials experiment [35] in which the influence of stimulus parameter on the visual gamma-band response was analyzed. The results showed that large stimuli evoked larger response amplitude. The authors explain this phenomenon by the assumption that larger stimuli activate larger cortical areas in the retinotopic visual cortices than smaller stimuli do [35]. Similar results were obtained by Ng [36]. In his study the size of examined stimuli ranged from 0.67 to 8.9 degrees (visual angle). The amplitude of SSVEP response grew in direct proportion with the size of the stimulus. We suspected that big and fair stimuli could make subject tired with time and the SSVEP could be reduced. Nevertheless, in case of big stimulus and white/yellow field neural response was stronger in compare to SSVEP evoked by more pleasant ones.

Inter-stimulus distance and shape in this study revealed no significant effect on SSVEP magnitude. We suspected that presence of the other flickering fields in the visual field could hinder focus on cued square and affect SSVEP response. The magnitude of this response is strongly modulated by attention [17]. In our experiment, all tested distances were within human visual field, so in every condition all four squares were perceived. The results showed that interfering flickering stimuli do not have any impact on tested potentials. A significant impact of distance was showed by Ng [36]. The most accurate stimuli were placed at a distance of more than 5 degrees apart. This study differs methodologically from our experiment. We directly measured increase in magnitude in SSVEP response, while Ng used a classifier.

The presence of a fixation point lowered the strength of SSVEP response, but the mean differences were insignificant. That was contrary to our research hypothesis, and the effect was significant only in the case of two frequencies (17 and 25 Hz). These results can be explained on the psychophysiological level by properties of the visual pathways. When a fixation point is present, a person focuses on the center of the stimulus. The center of the visual field is dominated by the PC pathway, which has low temporal resolution [37]. Therefore, focusing attention on the center may lower subject's sensitivity to flickering. This phenomenon can explain the inconsistencies with psychological theories.

## Conclusions

This experiment, to the best of our knowledge, was the first detailed study examining specific parameters of stimuli and their relation to SSVEP magnitude. We showed the significant impact of some features of flickering field on the amplitude of the response. Based on our results, it seems that the best stimuli for BCI systems should be as bright and large as possible. The significant advantage of such parameters is an increase of SSVEP response, but they have disadvantages as well. If large fields are used, it can limit the amount of command buttons available in the menu panel of BCI systems. Also, very bright hues can be tiring to use for long periods of time.

A robust optimization should also take into account the possible interactions between the different parameters of the stimuli; in this work varied each parameter separately, which relates to the assumption of the lack of interactions. However, testing this hypothesis directly would require a large amount of repetitions of the experiment with different combinations of parameters. On the other hand, the main assumption which drove the experimental design, as mentioned in the Introduction, was proximity to the real-world application of BCI, including a significant duration of each stimulation to simulate possible adaptation and fatigue. In such setup a complete search of the solution space would require up to dozens days of signal collection for each participant, which exceeded the possibilities and aims of this research.

Optimal selection of the parameters of SSVEP stimuli appears to be one of the major factors influencing the efficiency of SSVEP-based BCI systems. The results presented in this paper provide a solid foundation upon which studies on the usability of particular designs of SSVEP based BCIs can be planned. In particular, further research should take into consideration the contrast between stimulus and background, as well as response specificity and maybe also possible interactions between different stimuli features.
